# Endothelial activation and stress index (EASIX) in coronary artery disease: a simplified measure as a promising biomarker

**DOI:** 10.1007/s00392-024-02566-4

**Published:** 2024-11-13

**Authors:** Daniel Finke, Hauke Hund, Norbert Frey, Thomas Luft, Lorenz H. Lehmann

**Affiliations:** 1 Department of Cardiology, University Hospital Heidelberg, Heidelberg, Germany; 2 German Center for Cardiovascular Research (DZHK), Heidelberg, Mannheim, Germany; 3 Internal Medicine V, Hematology, Oncology and Rheumatology, Heidelberg University Hospital, Heidelberg, Germany; 4 German Cancer Research Center (DKFZ), Heidelberg, Germany

Sirs,

We appreciate our colleagues’ comments regarding our manuscript. While lactate dehydrogenase (LDH), creatinine, and platelet levels are not primarily used to investigate endothelial dysfunction, the score was originally developed as a marker for this purpose [1]. The concept of the study was to transfer a marker that was elaborated in hematological patient cohorts to patients with a leading cardiovascular disease, such as CAD. This interdisciplinary approach took advantage of the broad availability of the EASIX parameters.

LDH and creatinine rise and platelets drop in a coordinated way in post-transplant microangiopathies (TMA) as well as in the pathologically related atypical hemolytic uremic syndromes (aHUS) [2]. This observation was the incentive to combine them into a biomarker that yields continuous values. In other cohorts, EASIX correlated with serum levels of NT-proBNP [3], sCD141 (soluble thrombomodulin) [4], angiopoietin-2 [5, 6], interleukin-18 [4–7], and low IGF1 [7], all of which are markers for endothelial damage.

However, the association of EASIX with endothelial damage is still correlative, therefore a direct link to endothelial dysfunction is currently investigated in a prospective clinical study (NCT05502887).

Independently of a direct link to endothelial dysfunction, EASIX identifies patients at high risk of death within the following years, as presented in this study. The objection that many extrinsic and intrinsic factors influence the three EASIX parameters is true for any clinical situation and not specific for CAD. And yet, EASIX appears to be a common denominator for the outcome. Of note, the Cox multivariate models shown in the study are adjusted to confounding factors, such as diabetes, arterial hypertension, age, sex, high-grade coronary artery stenosis and heart failure. The distribution of these parameters is significantly different in the training and validation cohort. Still, EASIX independently predicted mortality in both cohorts.

This allows us to stratify the risk of mortality in many different patient cohorts and EASIX can be applied in settings in which determination of cardiac biomarkers, such as NT-proBNP and hs-cTnT, was not done in the clinical routine or is not accessible. We would like to emphasize that we did not attempt to question the value of or outperform these well-established markers in cardiac diseases.

**Fig. 1:**
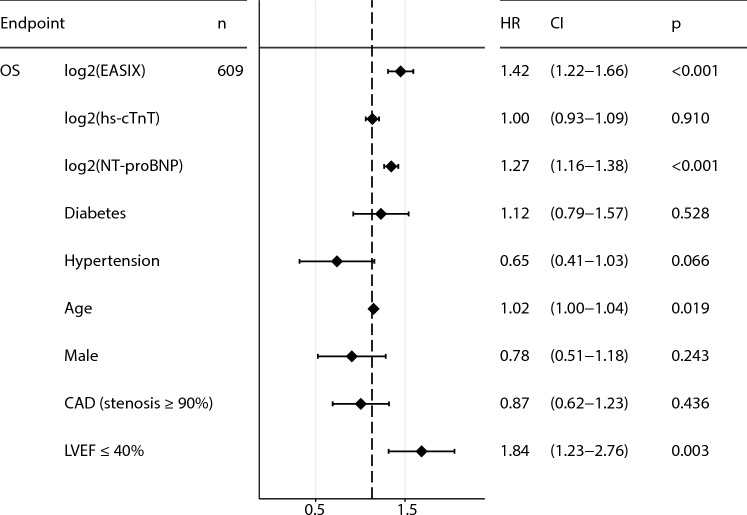
Multivariate analysis, including NT-proBNP and hs-cTnT. Forest plot representing Cox multivariate logistic regression model for 5-year overall mortality including log2(EASIX), log2(hs-cTnT), log2(NT-proBNP), diabetes, hypertension, age, the male sex, a high-grade coronary artery stenosis (≥ 90%) and reduced LVEF ≤ 40% as confounders in the validation cohort. EASIX was measured at median 174 days after coronary catheterization (CC) in the chronic phase. Hs-cTnT and NT-proBNP were measured at the time of CC. *CAD* coronary artery disease, *CI* confidence interval, *NT-proBNP* B-type natriuretic peptide, *HR* Hazard ratio, *hs-cTnT* cardiac high-sensitivity troponin T, *OS* overall survival

However, we re-evaluated a subgroup of patients (with available data) in a multivariate Cox regression analysis that survived 6 months after cardiac intervention and included the hs-cTnT and NT-proBNP levels measured (Fig. 1). EASIX preserves its predictive value.

Even though there is no clear clinical advice on further treatments regarding risk stratification via EASIX, this marker may help to identify patients at risk who need to be monitored in more detail. EASIX may be added to current risk stratification strategies that determine cardiological observation intervals in patients, suffering from CAD and/or oncological diseases.

Our colleague Rajakumar questioned the clinical value of the marker and correctly noted the lack of information on comedications. The value of EASIX in risk stratification can now be tested (even retrospectively) in clinical trials to specify the effects of any study medication in high and low-risk patients. Our own retrospective study on the use of statins in the context of alloSCT revealed that statins improved outcome in patients with intermediate pre-allo EASIX values [8].

Identification of a high-risk cohort with an increased risk of mortality by using easily available blood parameters makes EASIX a simple and attractive biomarker for a broad application. Translation in clinical practice and whether or not these patients will profit from a more aggressive intervention or surveillance need to be further evaluated in prospective studies.
